# Measuring diagnostic heterogeneity using text-mining of the lived experiences of patients

**DOI:** 10.1186/s12888-021-03044-1

**Published:** 2021-01-28

**Authors:** Chandril Chandan Ghosh, Duncan McVicar, Gavin Davidson, Ciaran Shannon

**Affiliations:** 1 Queen’s University Belfast, University Rd, Belfast, BT7 1NN UK; 2 IMPACT Research Centre, Northern Health and Social Care Trust, 60 Steeple Road, Antrim, BT41 2 RJ UK

**Keywords:** Diagnosis, Taxonomy, Heterogeneity, Lived experiences, Reliability

## Abstract

**Background:**

The diagnostic system is fundamental to any health discipline, including mental health, as it defines mental illness and helps inform possible treatment and prognosis. Thus, the procedure to estimate the reliability of such a system is of utmost importance. The current ways of measuring the reliability of the diagnostic system have limitations. In this study, we propose an alternative approach for verifying and measuring the reliability of the existing system.

**Methods:**

We perform Jaccard’s similarity index analysis between first person accounts of patients with the same disorder (in this case Major Depressive Disorder) and between those who received a diagnosis of a different disorder (in this case Bulimia Nervosa) to demonstrate that narratives, when suitably processed, are a rich source of data for this purpose. We then analyse 228 narratives of lived experiences from patients with mental disorders, using Python code script, to demonstrate that patients with the same diagnosis have very different illness experiences.

**Results:**

The results demonstrate that narratives are a statistically viable data resource which can distinguish between patients who receive different diagnostic labels. However, the similarity coefficients between 99.98% of narrative pairs, including for those with similar diagnoses, are low (< 0.3), indicating diagnostic Heterogeneity.

**Conclusions:**

The current study proposes an alternative approach to measuring diagnostic Heterogeneity of the categorical taxonomic systems (e.g. the Diagnostic and Statistical Manual, DSM). In doing so, we demonstrate the high Heterogeneity and limited reliability of the existing system using patients’ written narratives of their illness experiences as the only data source. Potential applications of these outputs are discussed in the context of healthcare management and mental health research.

## Background

A reliable categorical taxonomic system in the mental health context should offer a particular diagnostic category to people who share the same experiences and who can in turn be clearly distinguished from people with another diagnosis. In other words, it should be homogenous, i.e. it should feature within-group similarity and between-group dissimilarity. But the dominant taxonomic system of mental illness - the Diagnostic and Statistical Manual, or DSM - has been argued to be unreliable on the grounds of both Heterogeneity within, and comorbidity across, diagnostic categories [1, 44], suggesting that the current system of classification doesn’t fit well with people’s experiences. How such Heterogeneity is measured, however, is clearly critical to the assessment of DSM and other taxonomic systems. We assume that the DSM might have a limited distinguishing ability between patients with different diagnosis but not to the extent of being zero.

The first edition of the DSM [2] was primarily developed by a central committee of leading clinicians and researchers based on their own clinical experiences and their implicit understanding of the existing literature [8, 31]. Although later versions of the manual were constructed with more extensive documentation and with more explicit empirical support, concerns persisted over: criteria for revision (i.e. whether the decisions reflected the (potentially biased) perspectives of a small group of persons rather than systematic evidence), participation (i.e. inadequate opportunity for persons with divergent viewpoints to participate in the process), critical review (scepticism with the ability of persons to reach a fair, balanced, or optimal interpretation of inconclusive or inadequate research), and lack of comprehensive pilot testing (see [45]).

The DSM presents a classification of discrete, homogeneous disorders, but acknowledges that this structure cannot always be followed due to the overlap between diagnostic categories [1]. For example, a recent study identified 1030 unique symptom profiles in 3703 depressed patients [19], and it is questionable to tag all these profiles under “depression” (for example) and treat them with essentially the same intervention. The current system of mental illness classification does precisely that and is often criticised for putting two people with two unique profiles of symptoms under one diagnostic category. This is known as the “Problem of Heterogeneity” in the clinical diagnosis of mental illness. In other words, the way mental illness has been conceptualised – Major Depressive Disorder (MDD), Generalised Anxiety Disorder, Schizophrenia, and so forth - is problematic because it groups dissimilar symptom-profiles together.^1^ Such heterogeneous diagnoses lack treatment specificity, a clear clinical presentation, and precise diagnostic boundaries, and have high comorbidity rates and very low inter-rate reliability [17, 35].^2^ This limits advances in research on mental illness, treatment, how services are organised, and how people may view their experiences.

The measurement of Heterogeneity (and therefore unreliability), however, is controversial in its own right. Researchers have taken multiple approaches, each with its own limitations alongside limitations common to the different approaches.

First, theoretical assessments through reviews and perspective articles (e.g. [45, 46]) have pointed out issues concerning Heterogeneity such as the boundary disputes, and excessive diagnostic co-occurrence, although sometimes without explicitly using the term heterogeneity. The second set of studies have applied thematic analysis to qualitative data (e.g. [11]), including patient narratives (e.g. [1]), to code themes or patterns of meaning across the diagnostic categories from the chapters of the manuals (e.g. DSM-IV and − 5), with a particular focus on the Heterogeneity across the types of diagnostic categories.

Note that the study by Allsopp et al. [1] differs from the current study in several ways. Firstly, the source of data for that study was the five chapters (representing five disorders) of the DSM-5 and the no data from a human participant was involved (neither directly nor indirectly). Instead, that study analysed the chapters of the DSM-5 to infer Heterogeneity (i.e. analysing the chapters of a book) – manually (using the methods prescribed by [11]). In this current study, we do not analyse the DSM book, and our source of data was what the patients have written about their experiences. Furthermore, we did not rely on any particular disorder or a specific framework (e.g. DSM, or ICD). Instead, we focused on the symptoms, patients wrote about, in their narratives and analysed it using an automated process (eliminating human bias and enabling analysing a large dataset with speed).

A third approach has used survey designs or structured interviews to assess Heterogeneity through validation studies of disorder-specific symptomatic criteria. For example, a study aiming to validate the symptomatic criteria of MDD performed structured interviews on 1015 Caucasian twins diagnosed with MDD [27]. Logistic regression analyses revealed that different symptoms or groups of symptoms of MDD were associated with different clinical characteristics. Additionally, the study found a pattern of relationships between the diagnostic criteria of MDD with anxiety disorders and substance use disorders among several others, demonstrating comorbidity, i.e. symptoms overlapping across diagnostic categories. Another quantitative approach used latent-class growth-curve analysis (a modelling technique) on a prospective cohort database to study the life-course of depressive symptoms among older women over a period of almost 20 years [14]. The study identified four different trajectories of depressive symptoms, again suggesting Heterogeneity within specific diagnostic categories.

A fourth group of studies have tested reliability of diagnostic categories by measuring the degree to which two clinicians could independently agree on the presence or absence of selected DSM-5 diagnoses when the same patient was interviewed on separate occasions, in clinical settings, and evaluated with usual clinical interview methods (e.g. DSM-5 Field Trials: [39]). If the diagnostic criteria defining a disorder in a given group of patients cannot be assessed reliably by two or more clinicians – and the DSM-5 Field Trials demonstrated that 40% of diagnoses did not meet a relaxed cut-off for acceptable interrater reliability [39] – then the use of psychiatric diagnosis cannot be expected to aid treatment decisions [43]. Such studies have pointed out that co-occurrence among mental disorders (i.e. comorbidity of symptoms) is very common in both clinical and community samples [5, 7, 12, 22, 24, 34, 42].

While the first kind of studies (e.g. perspectives, opinions, and reviews) help us to clarify ideas and express alternative possibilities, they are theoretical in nature, so stop short of empirically demonstrating their arguments.

The second line of studies, where researchers use qualitative methodologies (e.g. thematic analysis, phenomenological studies) – do so manually, making the process of analysis prone to the researcher’s personal biases and idiosyncrasies, and potentially lacking rigour. Additionally, the collection of qualitative data of this nature is time-consuming and therefore is often carried out only at a small scale [4], limiting its generalisability. For example, a qualitative study conducted focus groups [36] to gather data about their perceptions about existing human immunodeficiency virus websites (for example). The transcripts were analysed using qualitative thematic analyses. The participants were 60 black female college students, and each focus group were conducted for 60 to 90 min. This method of the study had been quite popular until recently. While we acknowledge that analysing the transcripts enables researchers gain access to rich information (compared to surveys), we argue that they are expensive in terms of time and effort which often limits the number of participants that can be analysed. Furthermore, with reliance on human to analyse comes the possibilities of researcher biases, subjectivity, blind spots, and cognitive limitations (as reviewed by [28]). In this study, we opted for an alternative method of analysis, that is of automated text-mining to measure the Heterogeneity of a categorical system of diagnostic classification.

Thirdly, using surveys or structured interviews restricts the responses of the participant to only specific domains (while ignoring or neglecting others, such as related experiences, specific context, time, and so forth). Additionally, it is often criticised for under-representing/−covering specific groups of people from the target population (because of factors other than the ones being studied, e.g. lack of access to internet to fill the web survey or inability to travel to the researcher’s venue) and the ability of participants to select themselves for the survey, that is, self-selection bias [6]. For example, many people from the target population might not end up participating because of their apprehensions, negative attitude, or beliefs related to being a research participant. Thus, such survey or interview studies may cover only specific types of willing participants – and not others who might be willing to post online as anonymous writers. We overcome this limitation by using patients’ unstructured and unrestricted narratives about their lived experiences.

The fourth line of studies which employ clinicians to test if they arrive at the same diagnosis is prone to the failures of our decision-making shortcuts (heuristics), and the systematic and predictable errors in judgment that result from reliance on such heuristics, that is, cognitive biases (e.g. [9, 16, 23]). For example, a psychologist might suspect an abuse at home for a child patient demonstrating extreme fear because she (the psychologist) herself had a history of childhood abuse (availability bias).

A shared conceptual concern regarding these approaches is that they all seem to lean heavily on the assumption that corresponding constructs exist [10]. There are also concerns with the tools used to gather data for empirical studies. For example, many of these studies attempting to test Heterogeneity have used survey designs to collect data using questionnaires or inventories, or structured interviews. These self−/observer-report forms try to lump disparate symptoms to a sum-score (e.g. adding patients’ ratings on low mood, loss of pleasure, sleep disturbances, and so forth into a sum score of “total depression”), and into one category (e.g. “depression”), which is problematic for at least two reasons. First, the construct of depression is itself questionable [18]. Second, although using questionnaires/inventories/scales makes it possible to track invisible constructs like anxiety, depression, and distress in a manner that is economical (for the researcher or the clinician), it entails the loss of potentially valuable information by restricting responses of participants into forced choices (e.g. true or false, and ‘Rate on the scale of 1 to 5′).

The limitations of these current approaches have motivated a relatively novel approach to use text mining to study mental health. However, there are some serious concerns over the studies on that line. For example, a study explored mental health issues impacting college students using a corpus of news articles, foundation reports, and media stories [37]. The study attempted to do a cluster analysis to yield six themes in students’ mental health experiences in higher education (i.e., age, race, crime, student services, aftermath, victim). We argue that the finding from such studies represents more about what the popular media is discussing today than revealing facts about students’ mental health. Media coverage is often dependent on what the editor thinks will sell in the market, and we argue that studies using text mining on such media coverage suggests what the editors of the cited media prints. In this current study, we considered using data that comes directly from patients talking about their lived experiences with mental illness.

### Statement of problem

Each of the existing approaches to assessing Heterogeneity has its own limitations. The aforementioned methods used in the past to estimate the Heterogeneity of a diagnostic system raise methodological concerns, particularly in the domains of conceptualisation and usage of measurement tools. An alternate method to assess the Heterogeneity of the taxonomic system is therefore important because such methodological studies set the stage for ascertaining to what extent the current taxonomic systems will generate replicable clinical decisions.

### Aims and objectives

In this study, we aim to test two hypotheses. First, we aim to test if text-analysis of patients’ narratives is able to distinguish between patients’ experiences. We do so by examining whether we can distinguish between patients with different diagnostic categories, thus eliminating the possibility that dissimilarities between pairs of narratives reflect only the writing style of patients. In other words, our first aim is to test whether the homogeneity of illness experiences between the patients who received an identical diagnosis can be distinguished from the homogeneity of experiences for those who received different diagnoses using our proposed methodology. We do this by analysing 30 first-hand illness narratives of patients diagnosed with MDD and another 30 consisting of patients who received the diagnosis of Bulimia Nervosa (BN), using Jaccard’s Coefficient of Similarity index. We hypothesise that patients with the same diagnosis will have more symptoms in common, and thereby the similarity scores between narratives for pairs of people with the same diagnosis will be higher than for pairs of people with a different diagnosis. In doing so we introduce an innovative approach - text-mining of patients’ narratives - to the domain of mental health.

Our second aim is to contribute to the literature on the “Problem of Heterogeneity” by evaluating how similar patients’ experiences are, as expressed in their own narratives, across the board. The idea is to assess the degree of similarity between the illness narratives as a test for reliability, i.e. the extent to which patients with similar diagnoses expressed similar experiences. In doing so we aim to gain new insight into how consistent patients’ illness experiences are with the diagnosis they receive. As above, we hypothesise that narratives from people with the same diagnosis will be more similar (i.e. they will have a higher similarity coefficient) than they will be with people who received a different diagnosis. We estimate the degree of similarity using Jaccard’s Coefficient between each pair of illness narratives drawn from a sample of 228 narratives (228*227 = 51,756 pairs) - assessing the extent to which the current system of classification is heterogenous. We interpret a low coefficient value for the vast majority of the pairs as indicating high dissimilarity between patients irrespective of the diagnosis they received – thereby estimating the ability of the system to reliably categorise people. Our argument is that we can only reliably categorise people into groups (e.g. MDD, or BN) if we discover homogeneity – high similarity – between narratives within diagnostic group.

## Methods

This study uses a relatively novel text-mining approach for narrative analysis as a research methodology - to explore the extent of diagnostic Heterogeneity within patient’s symptomatic experiences.

### Data

In order to propose an alternative method of estimating diagnostic reliability we capture insights directly from the patients’ lived experiences (as opposed to the data gathered from surveys). In other words, we propose the use of the patients’ written narratives about their illness experience as an alternative to using survey data in assessing the “Problem of Heterogeneity.” The term ‘patient’ indicates people who self-reported that they received a psychiatric diagnosis (based on the DSM and ICD categories).

Accordingly, we analyse patients’ first-person narratives (*N* = 228) in the context of their lived illness experiences. These narratives consist of descriptions of how it is to live with mental illness and the nature of symptoms the patients experienced. This can potentially yield insights into how similar or dissimilar their experiences were among individuals with the same diagnoses, and whether people with diagnoses can be meaningfully sorted into similar groups. The study is based on personal accounts and data is collected from an online source (https://www.livejournal.com/). An example of another study that used livejournal to scrap the data (in relation to mental illness) would be that of Nguyen, Phung, Dao, Venkatesh and Berk [32]. LiveJournal provides a social platform for people of mutual interests to form and join communities and discuss about their medical conditions. Live Journal allows users to do so completely anonymously (and individuals do not have to attach their narrative with their name and photo) enabling a, perhaps, more supportive and less stigmatised environment.

The length of each narrative (before processing) varies but the average number of words is 586.5 (Standard Deviation, S.D. = 48.79). The longest narrative has 6087 words, while the shortest had at least 22 words. After processing (i.e. keeping only the symptoms/experiences), the patients expressed from two symptoms to 84 symptoms (average being 4.8 ~ 5 words with S.D. of 3.82 ~ 4).

These narratives have been written by people who were diagnosed with mental illness. While some of them write that they have recovered now, others are still experiencing mental illness. These published first-person narratives from patients are unlikely to encompass all types of patient, at all stages of their illness, and from all backgrounds. Instead, it seems likely that this approach may over-represent patients who are more expressive, out-going, literate (enough to write in the English language), insightful, and perhaps most importantly, who have recovered to the extent of being able to write their retrospective accounts. This might mean that important sub-groups of patients with psychopathology might be not be represented in this study, so we view this approach as potentially complementary to, rather than as a replacement for, existing approaches. However, the sample is representative in that it covers the majority of the diagnoses in DSM 5.

Data pre-processing (i.e. transforming the raw data, which may be contain spelling errors and filler words, into a cleaner format that is understandable by the computer) was done followed by creating a dictionary to retain only the relevant words for further analysis. The words in the dictionary were identified based on a manual screening of the word frequency table using domain knowledge, combined with reference to the DSM-5 [3], ICD-10 [47], and mental health websites (e.g. www.mind.org.uk).

There are several lexicon dictionaries available in the market. However, we did not find a readymade available dictionary that fits our philosophy and study goals. For example, Linguistic Inquiry and Word Count (LIWC, [41]) included words to capture attentional focus, emotionality, social relationships, thinking styles, and individual differences. EmoLex [30] comprised of a large lexicon of term–emotion associations. Likewise, the depression lexicon [15] which considered words related to depression and its symptoms based on the DSM and ICD. In this study, we wanted to study symptoms of psychopathology. While LIWC and EmoLex are important contributions to the literature and will be useful for further several psychological studies – it is not usable in this study which specifically relies on symptom analysis. Additionally, in this study we did not followed a particular manual (DSM/ICD). While we did refer to the DSM and ICD for gathering collections of words, but we also focused on manual scanning of the words the patients wrote about their mental ill health experiences – without specifying or restricting ourselves to a particular disorder or syndrome (e.g. depression). So, lexical dictionaries such the one cited above [15] was of limited use. Combined, due to unavailability of a suitable lexical dictionary for the purpose of this study, we build one of our own based on the manual search of what patients wrote and supplemented with more words from the DSM and ICD.

In building the lexicon for this study on psychopathology, we sought part inspiration from the method used by a previous study that focused just on depression [15]. We followed a similar approach, that is, combining DSM/ICD descriptions with patients’ report, but our focus was more general (not a particular disorder).

The first step in the process was the inclusion of the symptom words from both the gold-standard descriptions (i.e., DSM and ICD classification systems). In the second step, we analysed the first-hand accounts of the individuals themselves who experience the symptoms to ensure that the lexicon encompasses most of the domain. The following steps were taken to analyse the patient narratives:

1. To do so, we ran the narratives through the frequency-search program, which ranked every word from the 10 k + narratives in terms of the frequencies used.
2. Next, we did a manual scan of that ranked list of word frequencies to pick the words indicative and representative of a psychiatric symptom (e.g. “anxiety”) or experience (e.g. “hearing voices”). We included the relevant words, both the traditional and slang ways of symptom expression.
3. Redundant words (e.g. “the”, and “a”) were not included in the lexicon dictionary.

The output of the data pre-processing and the filtration process was a data with only the psychopathological symptoms. On that data, we estimated similarity metrics (e.g. Jaccard similarity index) on the symptoms, and word frequency search was conducted using Python 3 Code Script. To reiterate, we retained only symptoms and experiences based on the dictionary (for each user) to compute Jaccard similarities across users. Microsoft Excel was used for the heat-map visualisation (Table 1).

**Table 1:** Word frequency for the seven most frequent symptomatic words expressed in the pool of narratives and their respective weighted percentages

Word	Count	Weighted Percentage (%)
Depressive	7762	1.13
Anxiety	4661	0.68
Fears	1927	0.28
Hating	1909	0.28
suicides	1904	0.28
panics	1584	0.23
stressing	1457	0.21

The count gives the number of occurrences of the stemmed word in the sample of 228 narratives. The percentage gives the frequency of the word relative to the total words counted – to get an estimate of the frequency of specific words narrated in the context of the overall number of words used

### Analysis

**Fig. 1:**
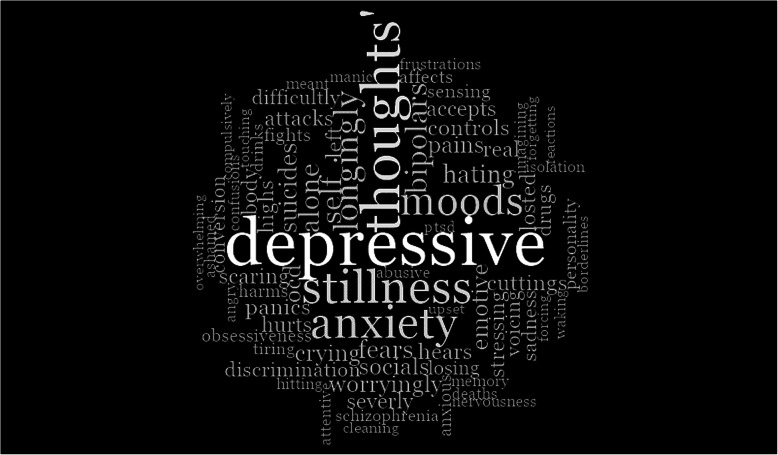
Word Cloud of reported experiences. *Note.* The word cloud is based on the frequency of the words expressed in the pool of narratives and represents the common themes in the narratives

We start with a simple descriptive analysis of the sample of narratives, focussing on the occurrence of relevant stemmed words (see Fig. 1 and Table 1). To test whether narratives can be used to distinguish between patients with different diagnoses, measures of similarity within- and across-diagnostic categories were estimated and compared. We selected the patients who reported that they had received a diagnosis of MDD and BN within their narratives from the 228 narratives in the database. These two disorders are ideal for this purpose because existing studies suggest they are relatively exclusive to each other, that is, they tend not to share symptoms [38], demonstrating a relatively high homogeneity and low comorbidity. Further, the absolute risk of receiving a diagnosis of an eating disorder (per 100 people) after a prior diagnosis of mood disorders was found to be less than 5% even after 15 years [38]. In contrast, comparing a highly comorbid combination of disorders such as eating disorder and anxiety disorder [13] or Mood Disorder with Neurotic Disorder [38] would likely result in an elevated similarity score across disorders, rendering it unclear if experiences of people diagnosed with the same disorder are relatively more similar than those who received a different diagnosis. In other words, as a test of narratives (as a valid data-source representing illness experiences), we estimated if people diagnosed with MDD had a higher similarity score on average (indicating similar illness experiences) compared to the ones who received a different diagnosis (i.e. BN, a form of Eating Disorder). If the narratives solely reflect writing style or other irrelevant factors, then we expect no significant difference in the average similarity scores within and between the two disorders.

We removed all the unnecessary words from the dataset (e.g. “the”, “is”, and “important”) except for the symptoms (e.g. low mood) and experiences (e.g. hearing voices). This is to reduce the volume of unnecessary words from the data and estimate similarity based on the symptomatic words only.

We then used Jaccard’s similarity index as the similarity metric, which measures the similarity between two nominal attributes by taking the intersection of both and dividing by their union. In other words, Jaccard similarity is the number of common attributes divided by the number of attributes that exist in at least one of the two objects. The rationale for choosing Jaccard similarity was associated with a potential problem with the nature of data: narratives often have repetitive words. Since we are comparing the symptomatic profile of two patients at a time, we do not want the number of times a specific symptom (by a person in his/her narrative) is mentioned to influence the similarity score. Instead, we want to estimate the similarity score by considering how many symptoms there are in common between two patients. Other similarity measures, such as cosine similarity, are affected by the frequency of word usage in the narratives. The rationale for disallowing the raw term-frequency to influence the similarity score was based on the belief that people’s writing style differ (e.g. some people tend to use the same words – in a repetitive manner). Using term-frequency might demonstrate how similarly two people are repetitive with their word-usage rather than the common symptoms they experience.

The similarity scores were estimated between all possible pairs of patients who received the diagnosis of either BN or MDD (*n* = 30*29 = 870 pairs in each case). For evaluating similarities between people across-disorders, all possible pairs of people where one person received a diagnosis of MDD (*n* = 30) and the other received a diagnosis of BN (*n* = 30) were analysed (*n* = 30*30 = 900). Results are presented in Table 2.

**Table 2:** Test of similarity of narratives obtained within and across clinical diagnoses

Diagnostic Categories	Major Depressive Disorder (MDD, ***n*** = 30)	Bulimia Nervosa (BN, ***n*** = 30)	t-test
**Major Depressive Disorder (*n*** **= 30)**	**0.120 (S.E. = 0.010)**	**0.030 (S.E. = 0.006)**	t(1768) = − 7.670, *p* = < 0.010
**Bulimia Nervosa (*n*** **= 30)**	**0.030 (S.E. = 0.006)**	**0.070 (S.E. = 0.009)**	t(1768) = − 6.390, *p* = < 0.010

The table depicts the average Jaccard’s coefficient and standard error of 60 patients - either with a diagnosis of Major Depressive Disorder, or Bulimia Nervosa. The t-test were conducted between the average Jaccard’s coefficient of 870 possible pairs (30*29) within disorders and the average for 900 pairs (30*30) of the patients across-disorder

To test the homogeneity of illness experiences between patients more generally, including among those who received an identical diagnosis, we repeated this exercise to estimate Jaccard’s coefficient between each pair of 228 (i.e. 228*227) narratives in the full dataset. Because existing studies find evidence of Heterogeneity we hypothesise that the similarity index between each pair of narratives will be low. Results are presented in Table 3.

**Table 3:** Top 10 narratives clustered by file similarity index indicated by Jaccard’s coefficient (in descending order)

Case A	Case B	Jaccard’s coefficient
Depression, Bipolar, Addiction, male	Bipolar, addiction, male	0.98556
Depression, Anxiety	Anxiety	0.823529
Depression	Depression, Anxiety	0.823529
Suicidality, Female	PTSD, Female	0.785441
Schizoaffective, female	Psychosis, female	0.693694
Postpartum depression, female	Depression, female	0.669355
Unspecified emotional disturbance, anger, fear, Male	Non-specific	0.184211
Suicide	Depression, Anxiety, ADHD	0.168675
depression and anxiety, PTSD, ADHD and borderline personality disorder	borderline personality disorder, Multiple more diagnoses	0.150442
Depression, Anxiety, ADHD	Bipolar 1, PTSD and panic disorder with agoraphobia, female, age18	0.144578
. . .		
Unspecified, male	Unspecified emotional disturbance, anger, fear, Male	0

Ethics approval for the study was obtained from the research ethics committee at the School of Management in Queens University Belfast.

## Results

### Descriptive statistics: describing the sample of 228 patients

Figure 1 presents the 75 most frequent words from the sample size (n) of 228 cleaned narratives. These are presented in varying font sizes, where the font sizes increase proportionately with the word’s frequency percentages.

From the word cloud, we can infer the nature of the words in the patient narrative sample. This provides a broad overview of the nature of illness people experienced in our sample pool. Note that the frequency of words was used here to analyse the most common words used by the patients whose narratives were analysed in this study. The aim is to identify possible themes in the database. The use of term frequencies here should not be confused with the above-mentioned rationale of not using term-frequencies to evaluate similarities between two narratives. Here the objective for using frequency of words (i.e. to explore the themes) is different from the one mentioned above (i.e. for evaluating similarity between two narratives). In the latter case we suggested that term frequency should not be used.

Table 1 presents counts and corresponding percentages for the seven most frequently occurring stemmed words. This kind of descriptive analysis could, potentially, help us to prioritise our focus towards the most frequently reported experiences for future intervention studies and perhaps even for training of mental healthcare professionals. Note the dominance of ‘depressive’ and ‘anxiety’, for example. Also note, however, that these kinds of narratives posted online in public forums may under-report certain experiences when compared with data-generated using other forms of data (e.g. in-person surveys, and interviewing) – which might suggest some of the less reported symptoms could still be prevalent (or at least existent). Such under-reporting could reflect stigma (e.g. in the case of sexual dysfunction), lack of understanding, or minimising the importance of something potentially significant (e.g. sleep), for example. In researcher-generated data such as surveys or interviews the participants are assured of confidentiality and anonymity for their responses, and there are no chances of being reprimanded or receiving inappropriate or offensive comments, unlike in online communities where anyone can make such comments in response to one’s post. It may be, therefore, that researcher-led surveys/interviews are less likely to be affected by stigma or fear of being judged than open public forums, although this is not clear.

### It is possible to distinguish people with different diagnoses using first-hand narratives of patients

If people who receive the same diagnosis are more likely to share illness experiences than those who receive a different diagnosis, then – if narratives are to be useful for our purposes here – we would expect to find evidence of this in their narratives. Table 2 presents the average similarity index of patients when compared pairwise with members who received the same diagnosis, and ones who received a different diagnosis. The similarity appears to be higher between the narratives of patients with the same diagnosis than between those with a different diagnosis (i.e. MDD and BN). We then use a one-tailed t-test on the independent sample means within-group and between-group to test formally if the text-mining-of-narratives approach is able to distinguish between within-group and between-group pairs. The 30*29 (= 870) pairs of narratives of patients who received the diagnosis of MDD (Mean, M = 0.12, Standard Error, S.E. = 0.01) compared to the 30*30 (= 900) pairs of narratives between patients who received the diagnosis of both MDD and BN, that is, MDD-BN (M = 0.03, S.E. = 0.00) demonstrated significant differences in their similarity scores, t(1768) = − 7.67, *p* = < 0.01. Likewise, a t-test between the narratives of people labelled with BN (M = 0.07, S.E. = 0.00) and MDD-BN (M = 0.03, S.E. = 0.00) shows a significant difference in their similarity scores, t(1768) = − 6.39, *p* = < 0.01. Combined, the results demonstrate statistically significant differences between within-diagnostic and between-diagnostic pairs. The suggestion is that narratives can indeed be used for the purposes of assessing the heterogeneity problem.

### A majority of the individuals (95%) when paired with narratives of all others had no similar partners at all – indicating high heterogeneity

Table 3 presents selected results of the similarity index estimation performed on all 228 patients’ narratives together. Specifically, Table 3 lists the top ten most similar narrative pairs, presenting the similarity coefficient in each case. The six pairs with most similar patient narratives were found to possess similar diagnoses (similarity range from 0.67 to 0.98), suggesting that 12 people (out of 228) shared their experiences closely with someone else in the sample, in each case someone with a similar diagnosis. Of course, we cannot rule out the possibility that these 12 narratives are for fewer than 12 people, i.e. that the same individual may have posted different narratives to more than one online forum that we scraped, or even the same forum at different times. If anything, however, this would strengthen our conclusion below. But since these high similarity pairs make up only a tiny proportion of our complete set of pairs (about 0.01%), we focus on the remaining 99.99% of the pairs which had low similarity. After the sixth pair of patients the similarity coefficient drops dramatically from 0.67 to 0.18, and it continues to fall to zero for the most dissimilar pairs (one example of which is given in the table). In other words, for 216 patients (out of 228, or 94.7%) their experiences (or at least their narratives of their experiences) are only weakly similar to any of the other narratives, including for peers who had received identical or similar diagnoses. Furthermore, the similarity index between 51,750 out of 51,756 pairs (99.98%), was equal to or below 0.30, and 252 pairs of patients had an absolute zero similarity. Peoples’ symptoms and experiences of mental illness, even when they share a diagnosis, are typically very different.

## Discussion

The current study used a text mining approach to explore the consistencies between the diagnoses people received and the similarity between their illness experiences as reported in their written narratives. This was based on our hypothesis that individuals with similar diagnoses would have more similar illness experiences than those with different diagnoses at least in some cases, but that overall the DSM would have a very weak ability to differentiate between people regardless of their diagnosis, rendering it limited in its scope for clinical applications.

Our results demonstrated the viability of using narratives as representations of illness experiences. Narratives of patients who received the same diagnosis had a higher similarity index on average than those who received different diagnoses, suggesting that the difference between similarity indexes is because of the diagnosis-related experiences (e.g. patients with MDD may experience low mood and loss of interest while patients with BN may experience purging and eating-related issues) instead of other aspects of the narratives (e.g. writing variation). However, the typical degree of similarity among these narrative pairs was still very low.

The core analysis revealed that the most similar narrative pairs do share the same diagnoses, but that very few narrative pairs have anything other than very lot similarity, even within diagnostic groups (only 6 pairs out of 51,756 had a similarity index of 0.67 and above, where the score ranges from 0 to 1; and 1 indicates 100% similar). We found that patients had dissimilar narratives more often than they were similar; about 99.98% of the computed pairs of narratives were only weakly similar, that is, with a similarity index at or below 0.18, with most far lower). This suggests that individuals’ narratives, and by extension, their experiences, are highly diverse, and therefore that diagnostic labels will struggle to categorise people into discrete categorical entities. This is in line with a recent report that showed two people could receive the same diagnosis without sharing any common symptoms, and that a considerable amount of Heterogeneity exists within the criteria of individual diagnoses in the majority of diagnoses in both DSM-IV-TR and DSM-5 (64 and 58.3% respectively, [33]). More generally, our findings provide further support to existing research using a wide variety of methods to demonstrate Heterogeneity within diagnostic categories of traditional taxonomies of mental ill-health.

Diagnostic Heterogeneity is a serious problem. Using the current DSM-5 criteria, two people can even make it possible to meet the diagnostic criteria for obsessive-compulsive personality disorder and share no diagnostic criteria [26]. The recent literature has realised the concern with Heterogeneity (among several others). In response, researchers are attempting to replace the DSM and the ICD for mental illnesses with newer approaches. One prominent example of such efforts is the Hierarchical Taxonomy of Psychopathology (HiTOP, [25]). The HiTOP (still under development) aims to integrate evidence from the existing literature on the organisation of psychopathology and sketch a system (of diagnosis) based on these data. It attempts to reduce heterogeneity within constructs by grouping related symptoms together and to assign unrelated symptoms to different dimensions.

The current study’s findings suggest that these novel text-mining approaches should be explored further to examine whether they help evaluate the diagnostic Heterogeneity in the HiTOP model and other emerging nosological approaches in general.

The use of patient narratives is innovative in this context; comorbidity is typically investigated by analysing symptoms, whereas here we analysed narratives encompassing symptoms but also life events, experiences with people, expectations, and so forth. The use of narratives is also revealing; while individuals might share symptoms, as a whole they tend to have very different experiences, and reductive quantitative research focussing on symptoms only might risk the loss of valuable qualifying and contextual information.

## Conclusion

Humans are unique in their life-course experiences. Patients might share some symptoms, but the way their lives are, with people, events, and interpretations of the world, likely differs extensively. The current study emphasises this by demonstrating the considerable diversity in patients’ narratives concerning their mental ill-health, including within diagnostic categories. Consequently, we propose that mental health researchers and clinicians consider not just patient symptoms but also their environments and life as a whole. For researchers, there is potentially useful information in such data. For clinicians, this is potentially important because two people might share a set of symptoms, but they might respond differently to the same treatment. Hence a personalised treatment plan may be more helpful than a one-size-fits-all approach to care services.

It is important to acknowledge the possibility that the patients’ narratives may have been informed or influenced by the current classification systems of mental health. For example, a patient reading about the diagnostic label online might develop certain expectations about the prognosis or symptom-package they should be experiencing based on the diagnostic label they received, which might lead a person to narrate his or her story in alignment with the DSM-based categories (read online) and their symptomatic experiences might be altered accordingly (e.g. I am experiencing X and Y, but online it says people with my kind of disorder experiences X, Y, Z, and A so I should be experiencing Z and A either at present without knowing or in upcoming future and I should keep an eye on any slightest signs of symptoms like Z and A). But the fact that we found high dissimilarity within group despite such possibilities of the news, social media, and the internet on patient’s perception of their disorder, suggests that any such effect is not strong enough to mask the underlying dissimilarity in patient experiences.

The study was also limited by a relatively small sample size (*n* = 228). Nonetheless, our results tentatively suggest that research findings based on a more detailed consideration of peoples’ experiences, using larger and more diverse samples, could perhaps help to generate outcomes (e.g. policy making and clinical services) that are more patient-centric; that are data-driven but not in an overly reductive manner.

In this study, we mentioned about pre-processing words (to address spelling errors). There is evidence that suggests a relationship between such linguistic characteristics and mental health [40]. Future studies could consider using this as an additional feature in their analyses instead of normalising such words (to better fit the goal of uncovering Heterogeneity). Further, the words and phrases (n-grams) differentially expressed across users in different groups could be evaluated statistically to obtain more insight. Both of these ideas were out of scope for the current study considering its goals and research questions. Hence are more likely to be better candidates for future investigation.

Our study had several merits over its predecessors, especially, the qualitative approaches. However, as within this study, the text-mining approach loose context. A judicious bargain would be to see this as a complementary approach to the traditional qualitative research. Furthermore, future studies using lived experiences can highlight why algorithms need to be examined with context.

A fundamental question for such further research (and ultimately practice) is whether we should seek to build a more robust taxonomic system, or whether we should move away from requiring a classification system in mental health by eliminating the necessity from the viewpoint of care. Our conjecture is that any attempt to group patient experiences will struggle to generate a convincing typology given individuals rarely seem to share subjective experiences; not only are patient narratives dissimilar within existing diagnostic categories, they are also – in contrast to existing studies that suggest high comorbidity among psychiatric disorders, and therefore the possibility of improved categorisation – dissimilar across diagnostic groups. Therefore any attempt to place an individual in a particular category risks repeating the mistakes of the traditional systems, i.e. imposition of a categorical nomenclature that leads to a substantial loss of information and diagnostic instability.

## Data Availability

The datasets used and/or analysed during the current study are available from the corresponding author on request.

## Abbreviations

DSM: Diagnostic and Statistical Manual of Mental Disorders
ICD: International Statistical Classification of Diseases
MDD: Major Depressive Disorder
BN: Bulimia Nervosa
S.D.: Standard Deviation
S.E.: Standard Error
LIWC: Linguistic Inquiry and Word Count
HiTOP: The Hierarchical Taxonomy Of Psychopathology

## Footnotes

1 One example of within-disorder heterogeneity is the almost 24,000 possible symptom combinations for panic disorder in DSM-5 [20]. Likewise, the DSM diagnosis of major depression is made when a patient has any 5 out of 9 symptoms. Thus, a patient who presents with psychomotor retardation, hypersomnia and gaining weight is scored the same as a patient who is agitated, sleeping poorly and is losing weight [27].

2 An example of co-morbidity across diagnostic groups is that a presentation of 270 million combinations of symptoms would meet the criteria for both Post-Traumatic Stress Disorder and Major Depressive Disorder [48]. A response to the apparent lack of homogeneity in DSM is an acceptance that an individual patient may simultaneously develop more than one “co-morbid” disorder [21]. However, the extent of co-occurring diagnoses found raises concern about “the validity of the diagnostic categories themselves – do these disorders constitute distinct clinical entities?” ([29] ,p.380).
